# Systematic Expression and Localization Profiling of Piezo2 in Rodent Pancreatic Islets

**DOI:** 10.3390/nu18132182

**Published:** 2026-07-05

**Authors:** Wenyi Jiang, Yumi Miyai, Haotian Zhang, Kensaku Fukunaga, Toshihiro Kobayashi, Hitomi Imachi, Takanobu Saheki, Takafumi Yoshimura, Rathana Ly, Junichiro Akimitsu, Masaki Ueno, Guoxing Zhang, Koji Murao

**Affiliations:** 1 Department of Endocrinology and Metabolism, Faculty of Medicine, Kagawa University, 1750-1, Miki-cho, Kita-gun, Kagawa 761-0793, Japan; s24d714@kagawa-u.ac.jp (H.Z.); fukunaga.kensaku@kagawa-u.ac.jp (K.F.); kobayashi.toshihiro@kagawa-u.ac.jp (T.K.); imachi.hitomi@kagawa-u.ac.jp (H.I.); saheki.takanobu@kagawa-u.ac.jp (T.S.); yoshimura.takafumi.j4@kagawa-u.ac.jp (T.Y.); s25d732@kagawa-u.ac.jp (R.L.); murao.koji@kagawa-u.ac.jp (K.M.); 2 Department of Pathology, Faculty of Medicine, Kagawa University, 1750-1, Miki-cho, Kita-gun, Kagawa 761-0793, Japan; miyai.yumi@kagawa-u.ac.jp (Y.M.); ueno.masaki@kagawa-u.ac.jp (M.U.); 3 Department of Ophthalmology, Faculty of Medicine, Kagawa University, 1750-1, Miki-cho, Kita-gun, Kagawa 761-0793, Japan; akimitsu.junichiro.h7@kagawa-u.ac.jp; 4 Department of Physiology, Medical College of Soochow University, 199 Ren-Ai Road, Dushu Lake Campus, Suzhou Industrial Park, Suzhou 215123, China; zhangguoxing@suda.edu.cn

**Keywords:** mechanosensitive ion channel, Piezo2, pancreatic beta cells, insulin secretion, metabolic stress

## Abstract

**Background**: Impaired insulin secretion by pancreatic beta cells drives chronic hyperglycemia, which characterizes type 2 diabetes mellitus. The mechanosensitive ion channel Piezo2 has been implicated in various physiological processes. However, its expression and functional role in pancreatic endocrine cells remain poorly understood. **Methods**: We investigated the expression, cellular localization, and potential functional significance of Piezo2 in the pancreatic islets of mice fed normal- and high-fat diets (HFD) using molecular, immunohistochemical, and immunofluorescence approaches. **Results**: Piezo2 mRNA and protein expression were detected in rat pancreatic tissue and the pancreatic beta cell line INS-1 via polymerase chain reaction and Western blotting analyses. Hematoxylin and eosin staining and histopathological analysis were performed to determine the localization of Piezo2, insulin, and glucagon in the islets of Langerhans from mouse pancreas. Immunofluorescence revealed that Piezo2 colocalized with insulin, glucagon, pancreatic polypeptide (PP, a pancreatic cell marker), and insulin/PP (suggesting *Ppy*-lineage beta cells). Piezo2 expression is significantly reduced in islets from HFD-fed mice and downregulated under high glucose conditions in INS-1 cells. Stretch stimulation, with or without D-GsMTx4 (a Piezo2-specific inhibitor), enhanced glucose-stimulated insulin secretion, whereas ruthenium red (a non-specific Piezo channel inhibitor) did not alter the response to high glucose. **Conclusions**: These findings demonstrate Piezo2 expression in pancreatic islets and suggest that it is enriched in beta cells and *Ppy*-lineage beta cells, minority in alpha cells and is responsive to metabolic stress. Although Piezo2 may contribute to beta-cell adaptation, its role in insulin secretion remains unclear.

## 1. Introduction

The pancreas plays a critical role in the endocrine system by maintaining glucose homeostasis through hormone secretion by several cell types located within the islets of Langerhans, primarily insulin (from beta cells, which lower blood glucose concentration) and glucagon (from alpha cells, which elevate blood glucose concentration). Type 2 diabetes mellitus (T2DM) is caused by a combination of insulin resistance in peripheral tissues and a relative deficiency in insulin secretion from pancreatic beta cells, which is largely attributed to impaired glucose-stimulated insulin secretion (GSIS), resulting in chronic hyperglycemia and impaired glucose homeostasis [1].

Mechanical stimuli are fundamental to a wide range of physiological processes including touch and pain sensations, vascular tone, and respiration [2]. These processes rely on mechanotransduction, the conversion of mechanical forces into electrical or biochemical signals, which is mediated primarily by mechanosensitive ion channels. In 2010, Coste et al. [2] identified a novel family of mechanically gated ion channels in mice consisting of the proteins Fam38a and Fam38b, later named Piezo1 and Piezo2, respectively, after the Greek word for “pressure”, based on their ability to respond to force, thereby establishing a molecular basis for force-induced ion flux. The corresponding human homologs were denoted as PIEZO1 and PIEZO2 [3].

Piezo1 and Piezo2 channels share ~40% sequence homology while maintaining a highly conserved overall structural architecture [4,5]. Piezo channels are characterized by a *C*-terminal ion permeation pathway and a functionally essential intracellular loop [6,7,8]. Subsequent cryoelectron microscopy revealed that Piezo channels assemble into a trimeric, three-bladed, propeller-like structure surrounding a central calcium-permeable pore [9]. This unique architecture enables piezoelectric channels to function as mechanotransduction sensors that convert membrane tension into ionic flux, thereby mediating physiological processes such as touch sensation, proprioception, and respiratory control [9,10,11].

Beyond their established roles in sensory biology and cardiovascular physiology, mechanotransduction mechanisms are increasingly recognized as important regulators of cellular signaling and metabolic processes. Emerging evidence indicates that Piezo1 contributes to metabolic regulation. Early studies have demonstrated that Piezo1 is expressed in the pancreatic beta cell line INS-1, where Piezo1-mediated Ca^2+^ influx potentiates glucose-stimulated insulin secretion in a manner similar to that of high glucose [12]. More recent evidence from Ye et al. [13] demonstrated that Piezo1 is expressed in both human and mouse pancreatic alpha and beta cells. Under diabetic conditions, Piezo1 undergoes glucose-induced nuclear translocation in beta cells, and pharmacological inhibition or genetic deletion of Piezo1 reduces intracellular Ca^2+^ elevation and GSIS. These findings suggest that altered mechanotransduction may participate in the pathogenesis of T2DM, and identify Piezo1 as a physiological regulator of insulin release.

In contrast, studies on Piezo2 have primarily focused on its role in sensory neurons, including mechanosensation, tactile perception, proprioception, and pain perception [3,14,15]. Piezo2 expression has also been reported in rat dorsal root ganglia, where it functions as a key mechanosensory channel [16,17]. However, the expression pattern and cellular localization of Piezo2 in the pancreas have not yet been systematically examined.

Therefore, the aim of the present study was to systematically characterize the expression and cellular distribution of Piezo2 in rodent pancreatic islets and investigate its functional role under metabolic stress and mechanostimulation.

## 2. Materials and Methods

### 2.1. Animals

Eight-week-old male mice (C57BL/6J) were purchased from Clea Japan, Inc. (Osaka, Japan) and housed at controlled temperature (25 °C) under a 12 h light/dark cycle in compliance with the Guide for Experimental Animal Research. Mice were allowed ad libitum access to a normal diet (control, 12.4 kcal % fat, *n* = 3) or high-fat diet (HFD, 45 kcal % fat, *n* = 3), along with water. They were killed after eight weeks of feeding, and pancreatic tissues were collected and fixed in 10% formalin for further examination.

Eight-week-old male Sprague-Dawley (SD) rats were purchased from Clea Japan, Inc. (Osaka, Japan) and maintained at room temperature under a 12-h light/dark cycle. The rats were allowed ad libitum access to normal chow and water. After four weeks of feeding, the rats were killed, and their pancreatic tissues were removed, rapidly frozen in liquid nitrogen, and stored at –80 °C. All experimental procedures involving animals were performed in accordance with the Guidelines for the Care and Use of Animals established by Kagawa University, and the protocol was approved by the Ethics Committee of 112.

### 2.2. Cell Culture

The rat insulinoma derived pancreatic beta cell line INS-1 cells were cultured in RPMI-1640 medium (FUJIFILM WAKO, Osaka, Japan) supplemented with 10% heat-inactivated fetal bovine serum (FBS; Hyclone, Wilmington, DE, USA), 50 μM 2-mercaptoethanol, 100 U/mL penicillin, and 100 μg/mL streptomycin under a 5% CO_2_ atmosphere at 37 °C. The culture medium was changed every two days, and the cells were subcultured once a week. To induce high glucose conditions, INS-1 cells were cultured in the above-mentioned medium containing 5.6, 11.2, 22.4 mM glucose for 7 days [18].

### 2.3. PCR

INS-1 cells and rat pancreatic tissues were lysed using TRI Reagent (Molecular Research Center, Cincinnati, OH, USA), and total RNA was isolated according to the manufacturer’s instructions. Five micrograms of RNA was reverse-transcribed into cDNA using a SuperScript II Reverse Transcriptase Kit (Invitrogen, Thermo Fisher Scientific, Waltham, MA, USA). Reverse transcription-PCR (RT-PCR) was performed using Piezo2 specific intron spanning primers (rat Piezo2-forward: 5′-GTATCACCATGCCAACCCCA-3′ and reverse 5′-GGCGACCATGGCATGAATTC-3′). GAPDH (rat GAPDH-forward: 5′-TGAACGGGAAGCTCACTGG-3′ and reverse: 5′-TCCACCACCCTGTTGCTGTA-3′) was used as a positive control for RT-PCR. The PCR amplification conditions were as follows: an initial denaturation step at 95 °C for 3 min, followed by 40 cycles of denaturation at 95 °C for 10 s, annealing at 60 °C for 30 s, and extension at 72 °C for 50 s. The final extension was performed at 72 °C for 5 min, after which the reactions were held at 4 °C. The PCR products were electrophoresed on 2% (*w*/*v*) agarose gel to verify the expected product size.

### 2.4. Hematoxylin and Eosin Staining

Formalin-fixed, paraffin-embedded blocks of mouse pancreatic tissue were cut into 4-μm-thick sections. The sections were deparaffinized and rehydrated, then immersed in Mayer’s hematoxylin for 15 min, followed by a wash in 40 °C water for 5 min. Finally, the sections were stained with eosin for 5 min.

### 2.5. Immunohistochemistry

Formalin-fixed, paraffin-embedded blocks of mouse pancreatic tissue were cut into 4-μm-thick sections. The sections were deparaffinized, rehydrated, and incubated in 3% hydrogen peroxide in methanol for 10 min to quench the endogenous peroxidase activity. Antigen retrieval was performed by heating the sections in 10 mM sodium citrate (pH 6) at 95 °C for 20 min, followed by blocking with 2% bovine serum albumin (BSA) in phosphate-buffered saline (PBS) for 10 min. The sections were incubated overnight at 4 °C with primary antibodies: rabbit anti-Piezo2 (1:50, Alomone Labs, Jerusalem, Israel), mouse anti-insulin (1:200, Santa Cruz, Dallas, TX, USA), or mouse anti-glucagon (1:200, Sigma-Aldrich, St. Louis, MO, USA). After washing with PBS, staining was performed using Simple Stain Rat MAX-PO (MULTI) (Nichirei Biosciences, Tokyo, Japan) and developed with 3,3′-diaminobenzidine tetrahydrochloride (DAB) at room temperature for 5–7 min. Sections were counterstained with hematoxylin and analyzed using an optical microscope (BX53; OLYMPUS, Tokyo, Japan).

### 2.6. Immunofluorescence Staining

Sections were deparaffinized and subjected to antigen retrieval by heating in 10 mM sodium citrate (pH 6) at 95 °C for 20 min, followed by blocking with 2% BSA. For double staining, the sections were incubated overnight at 4 °C with the following primary antibodies: rabbit anti-Piezo2 (1:200, Alomone Labs, Jerusalem, Israel), mouse anti-insulin (1:200, Santa Cruz, Dallas, TX, USA), mouse anti-glucagon (1:2000, Sigma-Aldrich, St. Louis, MO, USA), and mouse anti-pancreatic polypeptide (PP; 1:250, IBL, Gunma, Japan). After washing with PBS, the sections were incubated at room temperature for 60 min with Alexa Fluor^®^ 488-conjugated goat anti-rabbit IgG and Alexa Fluor^®^ 594-conjugated goat anti-mouse IgG secondary antibodies (1:1000, Thermo Fisher Scientific, Waltham, MA, USA). Sections were counterstained with 4′,6-diamidino-2-phenylindole (DAPI, Nacalai Tesque, Kyoto, Japan) for 5 min at room temperature. For triple-staining, sections were incubated overnight at 4 °C with primary antibodies: rabbit anti-Piezo2 (1:200, Alomone Labs, Jerusalem, Israel), guinea pig anti-insulin (1:50, Arigo Biolaboratories Corp., New Taipei City, Taiwan, China), and mouse anti-PP (1:250, IBL, Gunma, Japan). After washing with PBS, sections were incubated at room temperature for 60 min with Alexa Fluor^®^ 594-conjugated goat anti-rabbit IgG, Alexa Fluor^®^ 488-conjugated goat anti-guinea pig IgG and Alexa Fluor^®^ 647-conjugated goat anti-mouse IgG secondary antibodies (1:1000, Abcam, Cambridge, MA, USA). Fluorescent signals were visualized using a fluorescence microscope (BZ-X710; KEYENCE, Osaka, Japan).

### 2.7. Image Analysis

For quantification of Piezo2-positive endocrine cells, representative pancreatic islets were manually annotated in QuPath (version 0.7.0). Cell detection was performed using DAPI staining for nuclear identification. Mean cytoplasmic fluorescence intensities were measured for each detected cell. Positive staining thresholds were established based on the fluorescence intensity of background cells and were then applied uniformly to all images acquired under identical imaging conditions. The percentages of Piezo2-positive beta cells, alpha cells and PP cells were calculated as the number of Piezo2-positive/insulin-positive or Piezo2-positive/glucagon-positive or Piezo2-positive/PP-positive cells divided by the total number of insulin-positive or glucagon-positive or PP-positive cells, respectively. Because DAPI was not included in triple-labelling experiment, cell segmentation was not performed, regions positive for insulin or Piezo2 or PP were annotated as regions of interest (ROIs) instead. The percentages of Piezo2-positive area (%Area) within insulin-positive/PP-positive ROI, insulin-positive/PP-positive area within PP-positive or insulin-positive ROI were calculated as the Piezo2-positive area or insulin-positive/PP-positive area within endocrine ROI divided by the total endocrine marker-positive area.

Fluorescence intensity and colocalization analysis were performed using the Coloc 2 plugin in ImageJ/Fiji (ver. 1.54p, National Institutes of Health, Bethesda, MD, USA). Pearson’s correlation coefficient (Pearson’s R value) was calculated to evaluate the degree of spatial colocalization between fluorescence signals.

### 2.8. Western Blot Analysis

Thirty micrograms of protein was separated on a 4% sodium dodecyl sulfate-polyacrylamide gel and transferred onto polyvinylidene difluoride membranes for immunoblotting. Membranes were blocked overnight at 4 °C with 5% (*w*/*v*) skim milk in PBS containing 0.1% Tween 20. Blots were incubated overnight at 4 °C with primary antibodies: rabbit anti-Piezo2 (1:200, Alomone Labs, Jerusalem, Israel) or mouse anti-α-tubulin (1:1000, Cell Signaling Technology, Danvers, MA, USA), followed by incubation with HRP-conjugated goat-anti-rabbit (1:5000, WAKO, Japan) or goat-anti-mouse (1:5000, Bethyl Laboratories, Inc., Montgomery, TX, USA) secondary antibodies for 1 h at 4 °C. The membranes were washed three times with PBS-T for 10 min each, and the antigen–antibody complexes were visualized using an ECL substrate (GE Healthcare, Chicago, IL, USA). Protein bands were detected using a luminescent image analyzer (LAS-1000 Plus; Fuji Film, Japan). Western blot band intensities were quantified using ImageJ software (National Institutes of Health, USA). For each sample, the intensity of the Piezo2 band was normalized to the corresponding α-tubulin band intensity to correct for differences in protein loading. Relative protein expression levels were calculated as the ratio of Piezo2 to α-tubulin and expressed relative to the control group.

### 2.9. Stretch Stimulation and Insulin Secretion Measurement

INS-1 cells were cultured on gelatin-coated BioFlex^®^ 6-well culture plates (FLEXCELL International Corporation, Burlington, MA, USA) for at least one week before stretch stimulation, with culture medium replaced every two days. Cells were washed for 1 h at 37 °C with Krebs–Ringer bicarbonate (KRB) solution (120 mM NaCl, 5 mM KCl, 2.5 mM CaCl_2_, 1.1 mM MgCl_2_, 25 mM NaHCO_3_, and 0.1% BSA), followed by an additional 1 h wash with 3.3 mM glucose KRB solution. During the last 15 min of the wash period, the buffer was replaced with fresh KRB containing 5 μM D-GsMTx4 (MedChemExpress, Monmouth Junction, NJ, USA) or 30 μM ruthenium red (Cayman Chemical, Ann Arbor, MI, USA). After washing, fresh KRB buffer containing 3.3 mM or 16.7 mM glucose, with or without inhibitors, was added to the cells. The cells were then exposed to cyclic mechanical stretching using a Flexcell Tension System (FLEXCELL International Corporation, Burlington, MA, USA) for 1 h in an incubator. A heart (P)-wave-like stretch pattern was applied at a frequency of 60 cycles per minute (cpm) with a stretch magnitude ranging from 0% to 10% elongation. Mechanical deformation was uniformly delivered in both radial and circumferential directions. Supernatants were collected and stored at –80 °C until analysis. Insulin concentrations were measured using an enzyme-linked immunosorbent assay kit (FUJIFILM Wako Pure Chemical Corporation, Osaka, Japan), according to the manufacturer’s instructions.

### 2.10. Statistical Analysis

Data are expressed as mean ± standard error of the mean (S.E.M), as specified in each figure. Statistical analyses were performed using the GraphPad Prism software (ver. 8.0.2.263). Data normality was assessed using the Shapiro–Wilk test, and homogeneity of variance was evaluated using the Brown–Forsythe test prior to parametric analyses. Comparisons between the groups were conducted using an unpaired two-tailed Student’s *t*-test, one-way ANOVA, or two-way ANOVA, as appropriate. Statistical significance was set at *p* < 0.05. The sample sizes (*n*) are shown in the figure legends.

## 3. Results

### 3.1. Piezo2 Is Expressed in Rodent Pancreas

**Figure 1:**
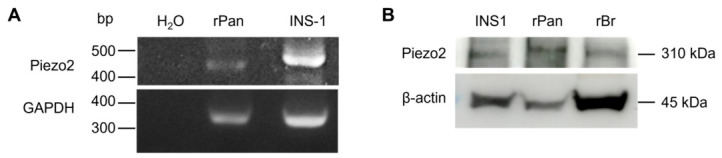
Piezo2 is expressed in rat pancreas and in INS-1 cells. (**A**) Representative agarose gel electrophoresis image showing 478 bp band for Piezo2 mRNA expression. H_2_O was used as negative control template for PCR. Expression of GAPDH (307 bp) was used as positive control for the RT-PCR. (bp: base pair). (**B**) Western blot analysis revealed the Piezo2 protein expression. β-actin was applied as loading control. rPan: rat pancreas; rBr: rat brain.

To determine whether Piezo2 is expressed in rat pancreas, particularly in pancreatic beta cells, specific primers for Piezo2 were used in PCR (Figure 1A). Distilled water served as the negative control and produced no detectable bands. Piezo2 was amplified from both rat pancreatic tissue and the rat pancreatic beta cell line INS-1, appearing as a 478 bp band in the representative agarose gel electrophoresis image. GAPDH was used as the positive control for RT-PCR. The specificity and sensitivity of the anti-Piezo2 antibody were confirmed by the manufacturer’s data and Shin’s recent study [19]. Western blot analysis confirmed Piezo2 protein expression in INS-1 cells and rat pancreatic tissues, with rat brain tissue serving as a positive control (Figure 1B).

### 3.2. Distribution of Piezo2 in Mouse Pancreatic Islets of Langerhans

**Figure 2:**
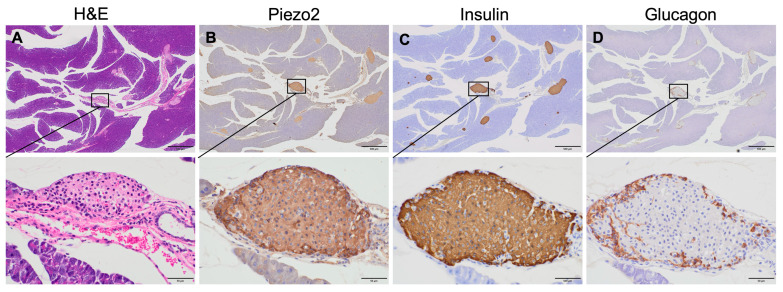
Immunohistochemical localization of Piezo2, Insulin and Glucagon in mouse pancreatic islets of Langerhans. (**A**) Representative hematoxylin and eosin (H&E) staining of mouse pancreatic tissue. Nuclei are stained blue-purple by hematoxylin, the cytoplasm and extracellular matrix are stained pink by eosin. Immunoperoxidase images of islets of Langerhans on adjacent sections showing immunoreactivity for (**B**) Piezo2, (**C**) insulin and (**D**) glucagon, respectively. Brown DAB staining indicates positive immunoreactivity, and nuclei are counterstained with hematoxylin (blue). Scale bars = 500 µm (upper panels), 50 µm (lower panels).

The histological morphology of the mouse pancreas was assessed by hematoxylin and eosin staining (Figure 2A), which revealed the islets of Langerhans as pale-stained, well-defined clusters of endocrine cells embedded within the darker-stained exocrine acinar tissue.

In human pancreatic islets, beta cells constitute the predominant cell type, accounting for approximately 50–70% of the total islet cell population, and are primarily localized within the central core of the islet. Alpha cells, representing the second most abundant population (approximately 24–40%), are predominantly distributed in the peripheral regions of the islet [20].

To determine the localization of Piezo2 in the mouse pancreas, single islets were analyzed by immunohistochemistry for Piezo2, insulin-secreting beta cells and glucagon-secreting alpha cells. Piezo2 staining was observed predominantly in the internal region of the islets, with high immunoreactivity in the peripheral cells (Figure 2B). Most cells within the islets showed insulin immunoreactivity, including peripheral cells, indicating the presence of beta cells (Figure 2C). As expected, the peripheral cells were specifically immunoreactive for glucagon, confirming the localization of alpha cells (Figure 2D).

### 3.3. Distribution of Piezo2 in Mouse Endocrine Cells

**Figure 3:**
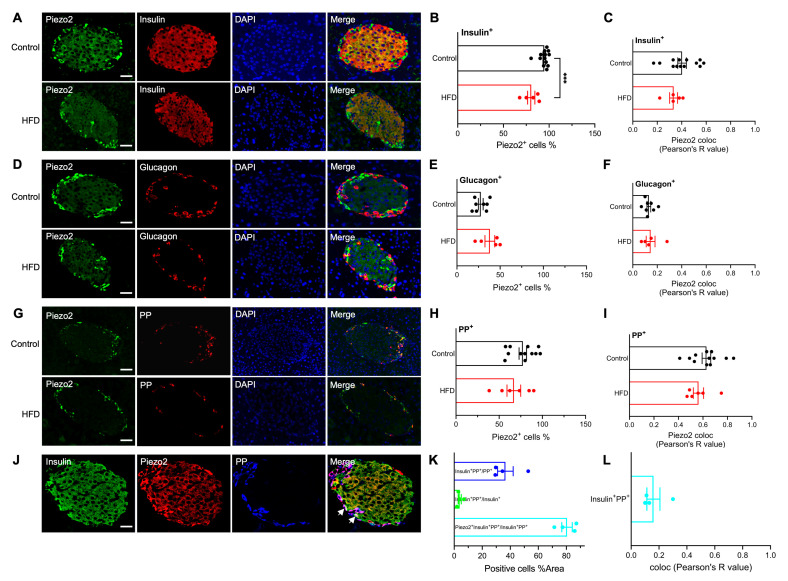
Distribution of Piezo2 in endocrine cells in mouse pancreatic islets of Langerhans. Immunofluorescence analysis of pancreatic islets from control and HFD-fed mice showing Piezo2 localization (green) with insulin-positive beta cells (red) (**A**), or glucagon-positive alpha cells (red) (**D**), or PP-positive PP cells (red) (**G**). Nuclei are counterstained with DAPI (blue). Areas of colocalization appear yellow in the merged images. Scale bars = 50 µm. The percentage of Piezo2-positive (% cell number) beta cells (**B**), alpha cells (**E**), PP cells (**H**). Colocalization of Piezo2 with beta cells (**C**), alpha cells (**F**) and PP cells (**I**). (**J**) Fluorescent triple labelling of insulin (green), Piezo2 (red) and PP (blue). Colocalizations of insulin^+^Piezo2^+^, insulin^+^PP^+^, Piezo2^+^PP^+^ appear yellow, cyan, magenta, respectively in merged image. White arrowheads indicate cells costaining insulin^+^Piezo2^+^PP^+^ appearing white. (**K**) The percentages of positive area (%Area). Blue column: Insulin^+^PP^+^/PP^+^, Insulin^+^PP^+^ area in PP cells; Green column: Insulin^+^PP^+^/Insulin^+^, Insulin^+^PP^+^ area in beta cells; Cyan column: Piezo2^+^Insulin^+^PP^+^/Insulin^+^PP^+^, Piezo2^+^Insulin^+^PP^+^ area in *Ppy*-lineage beta cells. (**L**) Colocalization of insulin^+^PP^+^. Scale bars = 50 µm. Each dot represents one pancreatic islet. Islets were isolated from three independent mice per group. Data are represented as mean ± S.E.M, *** *p* < 0.001 vs. the Control group (two-tailed unpaired *t*-test).

To determine whether Piezo2 was expressed in alpha and beta cells, immunofluorescence staining was performed on pancreatic sections from normal diet- (control) and HFD-fed mice. Piezo2 predominantly colocalized with insulin-positive beta cells in the central region of the islets (Figure 3A). Piezo2^+^ beta cells % was significantly lower in HFD-fed mice (*p* = 0.0006, 80.44% ± 4.01%) compared with control group (94.72% ± 1.45%) (Figure 3B). Colocalization was also observed between Piezo2 and glucagon in the islet periphery (Figure 3D); while no more than 40% alpha cells expressed Piezo2, no significant differences were observed between the control (27.64% ± 2.76%) and HFD group (38.10% ± 5.68%) (Figure 3E).

The pancreatic islets contain four main endocrine cell types. In addition to alpha, beta, and delta cells, PP cells (formerly known as gamma cells) secrete PPs to regulate both endocrine and exocrine functions [21], and are primarily located at the islet periphery and within the exocrine pancreas. Piezo2 localization relative to PP cells revealed a partial overlap between Piezo2 and PP signals at the islet periphery (Figure 3G) in 77.39% ± 4.31% control and 67.03% ± 7.97% HFD groups (Figure 3H).

Pearson’s correlation analysis revealed positive correlation between Piezo2 expression and insulin^+^ beta cells (Control: r = 0.40 ± 0.03; HFD: r = 0.33 ± 0.03, *p* = 0.2707) (Figure 3C), glucagon^+^ alpha cells (Control: r = 0.13 ± 0.02; HFD: 0.15 ± 0.04, *p* = 0.6747) (Figure 3F), PP^+^ cells (Control: r = 0.63 ± 0.04; HFD: r = 0.56 ± 0.04, *p* = 0.2837) (Figure 3I).

Recent lineage-tracing studies [22] have demonstrated that *Ppy*-expressing cells contribute not only to classic PP cells but also to approximately 12–15% of the total beta cells, predominantly at the islet periphery. Given that peripheral Piezo2 expression did not fully overlap with that in PP-positive cells, we hypothesized that Piezo2 is also present in *Ppy*-lineage beta cells, a minor beta cell subpopulation co-expressing PP and insulin. To test this hypothesis, triple immunofluorescence labelling for Piezo2, insulin, and PP was performed (Figure 3J). The analysis revealed 3.92% ± 1.09% insulin^+^ cells co-expressing PP, 36.55% ± 5.56% PP cells co-expressing insulin, of these insulin/PP double-positive cells, 80.46 ± 3.73% were positive for Piezo2 (Figure 3K), indicating that Piezo2 is highly enriched in insulin/PP co-expressing cells, which are considered *Ppy*-lineage beta cells. Correlation between insulin^+^ and PP^+^ cells was revealed by Pearson’s r = 0.16 ± 0.05 (Figure 3L). Nuclear translocation of Piezo2 was not observed in the control or HFD-fed mice.

### 3.4. Functional Analysis of Piezo2 in Pancreatic Beta Cells

**Figure 4:**
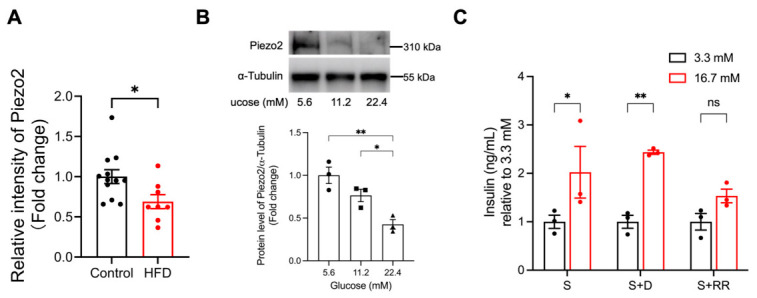
Functional analysis of Piezo2. (**A**) Quantification of fluorescence intensity of Piezo2 in Figure 3 (*n* = 12 and 8 islets from 3 mice in the control and HFD groups, respectively). Data are represented as mean ± S.E.M, * *p* < 0.05 vs. the Control group (two-tailed unpaired *t*-test). (**B**) Protein expression of piezo2 in INS-1 cells after 7 day culture in different concentrations of glucose, *n* = 3 as indicated. Data are represented as the mean ± S.E.M, * *p* < 0.05, ** *p* < 0.01. Statistical analysis was performed using one-way ANOVA multiple comparisons, followed by Tukey’s test. (**C**) Insulin secretion in INS-1 cells at the indicated glucose concentrations with stretch stimulation (S) for 1 h, with or without Piezo2-specific inhibitor D-GsMTx4 (D, 5 μM) or non-specific Piezo channel inhibitor ruthenium red (RR, 30 μM), *n* = 3 as indicated. Data are the mean ± S.E.M, * *p* < 0.05, ** *p* < 0.01, ns: not significant. Statistical significances were evaluated by 2-way ANOVA multiple comparisons, followed by Tukey’s test.

To assess whether Piezo2 expression differed in the pancreatic islets of control and HFD-fed mice, Piezo2 fluorescence intensity was quantified, as shown in Figure 3. Piezo2 expression was significantly reduced in HFD-fed mice compared to normal diet-fed (control) mice (*p* = 0.0249) (Figure 4A). To evaluate the potential functional role of Piezo2 in INS-1 cells, the cells were cultured under different glucose concentrations for 7 days. Piezo2 protein expression decreased progressively with increasing glucose concentrations (11.2 mM: 0.76 ± 0.07; 22.4 mM: 0.43 ± 0.06. ** *p* = 0.0043, * *p* = 0.0459) (Figure 4B), suggesting that Piezo2 may respond to metabolic stress. We hypothesized that Piezo2 is involved in the regulation of GSIS. INS-1 cells were then exposed to low (3.3 mM) or high (16.7 mM) glucose together with stretch stimulation (heart-wave pattern, 60 cpm) for 1 h, and insulin secretion was measured. Stretch stimulation significantly enhanced GSIS by 2.024-fold (*p* = 0.0131), and the specific inhibition of Piezo2 with D-GsMTx4 (specificity was confirmed by the manufacturer) produced a comparable increase in GSIS (2.437-fold, *p* = 0.0015). In contrast, the inhibition of both Piezo1 and Piezo2 channels with ruthenium red did not increase GSIS (*p* = 0.1554) (Figure 4C). These findings indicate that Piezo1 contributes to the regulation of GSIS, whereas pharmacological modulation of Piezo2 had no significant effect on GSIS in INS-1 cells.

## 4. Discussion

In this study, we systematically investigated the expression, localization, and functional relevance of Piezo2 in the pancreatic islets. Our findings demonstrated that Piezo2 is expressed in the islets of Langerhans, with preferential localization in beta cells, lower expression in a subset of alpha cells, and enrichment in peripheral endocrine cells, including PP cells and *Ppy*-lineage beta cells. Piezo2 expression was sensitive to metabolic stress, as the fluorescence intensity was significantly reduced in HFD-fed mice and downregulated under chronic high-glucose exposure. Functional analyses indicated that although mechanical stretching enhanced GSIS, pharmacological inhibition experiments suggested that Piezo2 may not play a major role in regulating GSIS. Collectively, these results provide the first characterization of Piezo2 expression in pancreatic islets and suggest that Piezo2 expression is responsive to metabolic stress, rather than directly controlling insulin secretion, although its functional significance remains to be determined.

A notable finding of this study was the distinct cellular distribution of Piezo2 compared to that of its homolog Piezo1 in pancreatic islets. Previous studies have shown that Piezo1 is widely expressed in both alpha and beta cells, and mediates mechanosensitive Ca^2+^ influx, thereby contributing to insulin secretion [13]. In contrast, our immunofluorescence analyses revealed that Piezo2 was predominantly localized in beta cells, while weaker expression was also detected in a subset of alpha cells, indicating a markedly different cellular distribution from Piezo1. Furthermore, Piezo1 has been reported to undergo nuclear translocation under certain mechanical or metabolic conditions [13], suggesting a potential role in transcriptional regulation or mechanosensitive signaling. The relatively limited expression of Piezo2 in alpha cells, together with the lack of detectable nuclear localization in our observations suggest that Piezo1 and Piezo2 perform distinct functions in islet biology. These differences support the hypothesis that Piezo1 and Piezo2 play non-redundant roles in islet biology. Consistent with the established role of Piezo1 in GSIS and beta cell Ca^2+^ signaling [13], recent studies have demonstrated that Piezo1-mediated mechanotransduction links extracellular matrix stiffness to altered islet Ca^2+^ dynamics and insulin secretory function [23]. Taken together, these findings suggest that Piezo channels are important mediators of islet mechanobiology. However, the physiological role of Piezo2 in the pancreatic endocrine cells remains largely unknown. Therefore, our findings identify Piezo2 as a previously underappreciated mechanosensitive channel in beta cells and provide a foundation for future studies investigating its contribution to islet function and metabolic diseases.

Another notable observation was the enrichment of Piezo2 in peripheral endocrine cells, including PP and *Ppy*-lineage beta cells. In addition, both the percentage of Piezo2^+^ beta cells and the fluorescence intensity of Piezo2 were significantly reduced in HFD-fed mice, indicating that Piezo2 expression is dynamically regulated under metabolic stress. *Ppy*-lineage beta cells are a distinct beta cell subpopulation [22] that emerge under metabolic stress and are characterized by reduced glucose responsiveness, decreased GLUT2 expression, and attenuated glucose-stimulated Ca^2+^ responses, which likely contribute to insufficient insulin secretion. The co-localization of Piezo2 with both PP and insulin indicates that Piezo2 is expressed in at least part of this population. However, the biological significance of this preferential expression pattern remains unclear because no direct functional evidence was obtained in the present study. The intense peripheral Piezo2 signal may reflect its known role in membrane tension sensing, potentially influencing processes such as cell volume regulation or paracrine communication [15].

Mechanical signals arising from extracellular matrix remodeling, vascular flow, or changes in islet architecture [24,25] are increasingly recognized as regulators of endocrine cell functions. In the present study, metabolic stress consistently reduced Piezo2 expression both in vivo and in vitro, as demonstrated by the decreased percentage of Piezo2^+^ beta cells and reduced Piezo2 fluorescence intensity in HFD-fed mice, together with downregulation of protein expression following chronic high-glucose exposure. These findings suggest that Piezo2 expression is responsive to metabolic stress. However, whether this reduction represents an adaptive response, contributes to beta cell dysfunction, or simply reflects altered cellular status cannot be determined from the current data. Given that Piezo2 functions as a mechanosensitive ion channel in multiple tissues, it will be important for future studies to determine whether altered Piezo2 expression influences mechanosensitive signaling in pancreatic endocrine cells.

Our functional data further indicated that Piezo2 may not play a major role in the acute regulation of GSIS under the experimental conditions examined. Mechanical stretching significantly enhanced insulin secretion in our experiments, which is consistent with previous reports showing that mechanotransduction can modulate GSIS [13]. However, pharmacological inhibition of Piezo2 did not significantly alter GSIS or the insulin secretory response to mechanical stretching. These findings indicate that Piezo2 is unlikely to be the principal mediator of stretch-induced insulin secretion in INS-1 cells. Nevertheless, because our conclusions were based solely on pharmacological approaches, the involvement of Piezo2 cannot be definitively excluded. Further studies employing genetic loss- and gain-of-function strategies in primary beta cells and pancreatic islets will be required to clarify the specific contribution of Piezo2 to beta cell mechanotransduction and insulin secretion. In contrast, previous studies have demonstrated that Piezo1 contributes to mechanically induced Ca^2+^ influx and insulin secretion in beta cells [12], suggesting that Piezo1 may play a more prominent role in these processes.

Accumulating evidence indicates that Piezo2 influences systemic metabolic regulation. Piezo2 is a well-characterized mechanosensitive ion channel involved in multiple physiological processes including tactile sensation [26], proprioception [27], pain perception [28,29], respiration [30], and urinary function [31]. Mutations in PIEZO2 are associated with several human disorders, such as distal arthrogryposis, Gordon syndrome, and Marden–Walker syndrome [32,33]. Recent studies revealed sex-dependent differences in Piezo2 expression and function in sensory neurons, with female mice exhibiting higher Piezo2 expression than male mice [34]. Whether a similar sexual dimorphism exists in pancreatic islets remains unknown and warrants further investigation. More recently, the deletion of Piezo2 in sensory neurons has been reported to enhance insulin sensitivity and improve glucose tolerance in mice [35], suggesting that Piezo2 participates in the regulation of whole-body energy metabolism. Together with the expression pattern observed in the present study, these reports raise the possibility that Piezo2 may participate in metabolic regulation through multiple tissues. Given the established involvement of PIEZO2 in sensory neuron function and the emerging evidence linking Piezo2 signaling to glucose homeostasis, it is conceivable that altered Piezo2 activity may contribute to diabetes-associated dysfunction. Further studies are required to determine whether Piezo2 expression or function is altered in diabetic islets, and whether such changes participate in the pathogenesis of diabetes or its complications.

This study had several limitations. First, the functional role of Piezo2 was evaluated primarily using pharmacological inhibition without complementary genetic loss- or gain-of-function approaches. Therefore, the present study cannot establish a causal role for Piezo2 in pancreatic beta cell physiology, and future studies using genetic models will be required. Second, although antibody specificity was supported by the manufacturer’s validation, blocking peptide experiments, and previous reports, independent validation using Piezo2-deficient tissues or genetic approaches was not performed and therefore remains a limitation of the present study. Third, the functional analyses were performed exclusively in INS-1 cells, which may not fully recapitulate the physiological characteristics of primary pancreatic beta cells. The sample size used for functional assays was also limited. Therefore, the effects of Piezo2 modulation on insulin secretion should be considered in future studies. Further studies using isolated pancreatic islets, primary beta cells, and larger cohorts are required to validate and extend these findings. Finally, although our data suggest expression of Piezo2 in *Ppy*-lineage beta cells, lineage-tracing approaches are required to definitively confirm its distribution within specific endocrine cell subpopulations.

## 5. Conclusions

In conclusion, our study identified Piezo2 as a mechanosensitive ion channel expressed in pancreatic endocrine cells with a distribution pattern distinct from that of Piezo1. Piezo2 is enriched in beta cells and *Ppy*-lineage beta cells and is responsive to metabolic stress, indicating that Piezo2 expression is dynamically regulated under metabolic stress. Although pharmacological modulation of Piezo2 did not significantly affect GSIS under the conditions tested, these findings provide the first descriptive characterization of Piezo2 expression in pancreatic endocrine cells and provide a foundation for future studies investigating the role of Piezo2 in beta cell biology and metabolic diseases.

## Data Availability

The original contributions of this study are included in the article. Further inquiries can be directed to the corresponding authors.
